# The relationship between potentially traumatic or stressful events, HIV infection and neurocognitive impairment (NCI): a systematic review of observational epidemiological studies

**DOI:** 10.1080/20008198.2020.1781432

**Published:** 2020-08-13

**Authors:** G. Spies, S. Mall, H. Wieler, L. Masilela, E. Castelon Konkiewitz, S. Seedat

**Affiliations:** a DST/NRF South African Research Chair in PTSD, Department of Psychiatry, Faculty of Medicine and Health Sciences, Stellenbosch University, Stellenbosch, South Africa; b Division of Epidemiology and Biostatistics, School of Public Health, Faculty of Health Sciences, University of the Witwatersrand, Johannesburg, South Africa; c Faculdade de Ciências Médicas e da Saúde, Universidade Federal da Grande Dourados, Dourados, Brasil

**Keywords:** HIV, PLWHA, neurocognitive impairment (NCI), cognitive impairment, traumatic events, stress, PTSD, VIH, PLWHA, Deterioro neurocognitivo (NCI), Deterioro cognitivo, Eventos traumáticos, Estrés, TEPT, HIV, PLWHA, 神经认知障碍 (NCI), 认知障碍, 创伤事件, 应激, PTSD, • HIV and the experience of a traumatic event can have a negative impact on brain functioning.• This synthesis of evidence shows that people living with HIV who have endured a trauma at any point in their lives are more likely to have trouble remembering, paying attention and/or multitasking.

## Abstract

**Background:**

HIV/AIDS and potentially traumatic events (PTEs) or stressful life events (SLEs) and/or PTSD are independently associated with neurocognitive impairment (NCI). Literature suggests that HIV and PTE/SLE exposure independently and consistently affect various domains of cognition including language ability, working memory and psychomotor speed. There are limited data on the interaction between HIV infection and PTEs and their combined effect on NCI.

**Objective:**

In this systematic review, we synthesise evidence for the combined effect of HIV infection and PTEs and SLEs and/or post-traumatic stress disorder (PTSD) on NCI of people living with HIV/AIDS (PLWHA) from high-, middle- and low- income countries.

**Method:**

Our inclusion criteria were observational epidemiological studies (case-control, cohort and cross-sectional designs) that investigated the interaction of HIV infection, PTEs and SLEs and/or PTSD and specifically their combined effect on NCI in adults. We searched a number of electronic databases including Pubmed/Medline, PsycINFO, Scopus and Global Health using the search terms: cognition, HIV/AIDS, observational studies, trauma and permutations thereof.

**Results:**

Fifteen studies were included in the review, of which the majority were conducted in high-income countries. Ten of the fifteen studies were conducted in the United States of America (USA) and five in South Africa. Seven of these focused on early life stress/childhood trauma. The remaining studies assessed adult-onset PTEs and SLEs only. Eight studies included women only. Overall, the studies suggest that PTE and SLE exposure and/or PTSD are a significant risk factor for NCI in adults living with HIV, with impairments in memory and executive functions being the most likely consequence of PTE and SLE exposure.

**Conclusion:**

These findings highlight the need for trauma screening and for the integration of trauma-focused interventions in HIV care to improve outcomes.

## Abstract (es)

**Antecedentes**: El VIH/SIDA y los eventos potencialmente traumáticos (PTEs) o los eventos estresantes de la vida (SLEs) y/o TEPT se asocian independientemente con el deterioro neurocognitivo (NCI). La literatura sugiere que la exposición al VIH, PTE y SLE afecta de manera independiente y consistente varios dominios de la cognición, incluida la capacidad del lenguaje, la memoria de trabajo y la velocidad psicomotora. Hay datos limitados sobre la interacción entre la infección por VIH y los PTE, y su efecto combinado sobre el NCI.

**Objetivo**: En esta revisión sistemática sintetizamos evidencia del efecto combinado de la infección por VIH, PTEs y SLEs, y/o TEPT en el NCI de personas que viven con VIH/SIDA (PLWHA) en países de ingresos altos, medios y bajos.

**Método**: Nuestros criterios de inclusión fueron estudios epidemiológicos observacionales (diseño de caso-control, cohortes y diseños transversales) que investigaron la interacción de la infección por VIH, PTEs y SLEs y/o TEPT, y específicamente su efecto combinado sobre el NCI en adultos. Se realizaron búsquedas en varias bases de datos electrónicas, que incluyeron a Pubmed/Medline, PsycINFO, Scopus y Global Health, utilizando los términos de búsqueda: cognición, VIH/SIDA, estudios de observación, trauma y permutaciones de los mismos.

**Resultados**: Quince estudios se incluyeron en la revisión, de los cuales la mayoría se realizaron en países de altos ingresos. Diez de los quince estudios fueron realizados en los Estados Unidos de América (EE.UU.) y cinco en Sudáfrica. Siete de éstos se centraron en el estrés de la vida temprana/trauma infantil. Los estudios restantes evaluaron PTEs y SLEs cuya aparición fue en la vida adulta solamente. Ocho estudios incluyeron sólo mujeres. En general, los estudios sugieren que la exposición a PTE y SLE y/o TEPT es un factor de riesgo significativo para NCI en adultos que viven con VIH, con el deterioro en la memoria y las funciones ejecutivas como la consecuencia más probable de la exposición a PTE y SLE.

**Conclusión**: Estos hallazgos resaltan la necesidad de la detección de traumas y la integración de intervenciones centradas en el trauma en la atención del VIH para mejorar sus resultados.

## Abstract (zh)

**背景**: HIV/AIDS和潜在创伤事件 (PTE) 或应激性生活事件 (SLE) 和/或PTSD与神经认知障碍 (NCI) 独立相关。文献表明, HIV, PTE和SLE暴露独立且持续地影响认知的各个领域, 包括语言能力, 工作记忆和精神运动速度。关于HIV感染与PTE之间的相互作用及其对NCI的综合影响的数据有限。

**目的**: 在本系统综述中, 我们综合了HIV感染, PTE和SLE和/或创伤后应激障碍 (PTSD) 对高, 中, 低收入国家 HIV/AIDS携带者(PLWHA)NCI的综合效应的证据。

**方法**: 我们的纳入标准是观察流行病学研究 (病例对照, 队列研究和横断面设计), 且考查了HIV感染, PTE和SLE和/或PTSD的交互作用, 特别是它们对成人NCI的综合效应。我们在包括Pubmed/Medline, PsycINFO, Scopus和Global Health的众多电子数据库中, 使用以下搜索词进行搜索:‘认知’, ‘HIV/AIDS’, ‘观察性研究’, ‘创伤’及其排列。

**结果**: 15项研究被纳入综述, 其中大多数在高收入国家进行。 15项研究中, 10项在美国 (USA) 进行, 5项在南非进行。其中有7项关注生早期生活应激/童年创伤。其余研究仅评估成年后发作型PTE和SLE。8项研究仅包括女性。总体而言, 研究表明PTE和SLE暴露和/或PTSD是HIV成年携带者NCI的显著风险因素, 记忆和执行功能损伤最有可能由PTE和SLE暴露导致。

**结论**: 这些发现强调了创伤筛查以及在HIV护理中整合聚焦创伤干预措施的必要性, 以改善干预结果。

## 1. Background

Advances in the treatment of HIV have dramatically improved survival rates. HIV has transformed from an acute terminal disease to a chronic pharmacologically-managed condition through increased access to combination antiretroviral therapy (cART) around the globe. Nevertheless, HIV infection is associated with a range of sequelae including neurocognitive impairment (NCI). The neuropathogenesis of NCI is complex and is characterized by events such as oxidative stress, neuroinflammation, synaptic pruning and neuronal death (Kumar et al., 2009; Valcour et al., 2012; Williams, Zulu, Stein, Joska, & Naude, 2020). HIV crosses the blood brain barrier (BBB) early in the course of infection. There is considerable evidence that NCI related to HIV has a variable clinical trajectory (Alford & Vera, 2018; Habib et al., 2013; Heaton et al., 2015; Rubin & Maki, 2019a). Symptoms of NCI in people living with HIV/AIDS (PLWHA) are generally on a spectrum ranging from asymptomatic neurocognitive impairment to mild cognitive impairment to HIV associated dementia (Antinori et al., 2007). The range of dysfunctions include mental slowing, memory loss, and difficulties in complex tasks, motor disorders, and behavioural abnormalities (Simioni et al., 2010). Studies suggest that between 30 and 50% of PLWHA experience mild forms of NCI. Data from the United States of America (USA) suggest that approximately 2% of PLWHA experience a more serious form of NCI, HIV associated dementia (Heaton et al., 2010). Today, while the incidence of the most severe phenotype, HIV-associated dementia has declined, subtle forms of the disease such as asymptomatic neurocognitive impairment persist despite patients being on cART (Ambrosius, Gold, Chan, & Faissner, 2019). This may, in part, be attributed to the limited penetration of the BBB by certain antiretrovirals (e.g. HIV protease inhibitors, nucleoside analogues). Evidence of the effectiveness of BBB penetration of antiretrovirals on cognition is equivocal, with higher *and* lower CNS (central nervous system) penetration effectiveness (CPE) of antiretrovirals associated with cognitive improvements, as well a few studies finding no association between CPE and cognitive benefits (Yuan & Kaul, 2019).

NCI in PLWHA has a complex aetiology and it is likely that a number of risk factors may interact with the HIV infection to predispose its onset (Heaton et al., 2015). A number of studies from both high and low to middle-income countries using neuroimaging and epidemiological techniques have examined additional factors potentially associated with NCI in PLWHA. These include potentially traumatic events (PTEs), and post-traumatic stress disorder [PTSD] (a potential consequence of PTEs) (Kessler et al., 2014; McLaughlin et al., 2017; Scott et al., 2018), which have also been associated with NCI in HIV-negative samples (Jelinek et al., 2006; Lagarde, Doyon, & Brunet, 2010). PTEs during childhood, particularly neglect, are associated with decline in memory, executive function, and processing speed (Malan-Muller et al., 2013; Spies, Ahmed-Leitao, Fennema-Notestine, Cherner, & Seedat, 2016). PTEs encompass a broad spectrum of exposures including sexual, physical, and emotional abuse during early life, adolescence, or adulthood. PTEs have been found to be highly prevalent in PLWHA (Brief et al., 2004; Decker et al., 2016; Machtinger, Wilson, Haberer, & Weiss, 2012). These PTEs have also been associated with a range of additional psychopathologies, most frequently PTSD and depression (Spies et al., 2012). A recent systematic review by Rubin and Maki examined the relationship between depression and NCI in PLWHA and found that depression contributed to impairments particularly in the domains of executive function, processing speed, learning, and motor function (Rubin & Maki, 2019b). A broad body of evidence links PTSD to impairments in multiple cognitive systems, including processing speed, learning, memory, and executive function (Aupperle, Melrose, Stein, & Paulus, 2012; Qureshi et al., 2011; Schuitevoerder et al., 2013; Scott et al., 2015; Sumner et al., 2017). In a meta-analysis based on data from 60 studies totalling 4,108 participants, including 1,779 with PTSD, 1,446 trauma-exposed comparison participants, and 895 healthy comparison participants without trauma exposure, significant neurocognitive effects were associated with PTSD (Scott et al., 2015).

While literature suggests that HIV infection, PTEs, and/or PTSD independently affect various domains of cognition including language ability, working memory, and psychomotor speed (Grant, 2008; Heaton et al., 2010; Tomoda et al., 2011), few studies have examined the combined effect of PTEs, PTSD, and/HIV infection on NCI (i.e. HIV+PTEs and/or PTSD on NCI). Spies and colleagues who work in South Africa, against the backdrop of a substantial HIV epidemic, examined the relationship between HIV, trauma and NCI in women, both as independent and combined exposures. They found that PTEs were significantly associated with memory impairment in women living with HIV (Spies, Fennema-Notestine, Archibald, Cherner, & Seedat, 2012). Findings from the Women’s Inter-Agency HIV Study (WIHS) indicated an interaction between psychological risk factors, including perceived stress, anxiety, post-traumatic stress, and depressive symptoms, and NCI, such that perceived stress and anxiety were more strongly associated with deficits in learning and memory among women living with HIV compared to their uninfected counterparts (Maki et al., 2015). Specifically in this study, depressive symptoms were associated with a lower level of cognitive performance (Maki et al., 2015). The findings suggest that the neurobiological effects of psychological risk factors, such as stress on cognition, may be different in HIV-positive and HIV-negative women, with putative mechanistic links to stress responsivity and immune function (Rubin et al., 2017). Stress, PTSD, and depression are immunomodulatory and influence the immune response in women infected with HIV. However, stress may have a stronger influence than depression given that stress is more strongly associated with regulatory mechanisms necessary to maintain immune cell homoeostasis (Rehm & Konkle-Parker, 2017), in turn impacting brain homoeostasis (de Groot & Burgas, 2015). Despite the gradual emergence of a small body of research examining the combined effect of PTEs and SLEs and NCI, to our knowledge no review has synthesised observational epidemiological studies of PTEs or SLEs and/or PTSD and NCI in PLWHA. For this review, we sought to synthesise findings from derived from high-, middle-, and low- income countries to contribute to the growing body of research exploring the relationship between PTEs and SLEs, and/or PTSD, HIV infection and NCI.

## 2. Methods

The process as outlined in PRISMA was adhered to (Moher, Liberati, Tetzlaff, & Altman, 2009).

### 2.1. Inclusion and exclusion criteria

Eligible studies had to be in English and include adults (18 years and older) only. There was no limit on the date of publication. We included observational epidemiological studies (i.e. cross-sectional, cohort, or case-control studies) that examine the relationship between PTEs and/or PTSD and NCI in PLWHA. Intervention studies were also excluded. In this instance, trauma was broadly defined as traumatic or stressful events (PTEs and SLEs) during childhood and/or adulthood. PTEs included both early life and adult-onset traumatic events that met Criterion A for a traumatic event for PTSD as well as other stressful life events (SLEs, e.g. economic hardship, food insecurity) and traumatic brain injury. For a study to be included, NCI had to be measured by a full neuropsychological battery. Studies using self-reported measures of NCI were excluded unless they included z-scores based on published normative data. Moreover, studies using screening instruments only for cognitive impairment (e.g. the (International) HIV Dementia Scale and the Montreal Cognitive Assessment) were excluded. Inclusion and exclusion criteria are presented in Table 1.

**Table 1.** Study inclusion and exclusion criteria.

Inclusion criteria	Exclusion criteria
Studies investigating neurocognitive impairment in the context of HIV and potentially traumatic or stressful events, specifically the combined impact of trauma on cognitive impairment in the context of HIV	Studies not investigating the impact of potentially traumatic or stressful events on cognitive impairment in the context of HIV
Adult samples (≥18)	Youth samples (<18)
Observational studies	Randomised controlled trials, quasi-experimental study designs and qualitative studies.
English language studies only	Non-English language studies
NCI measured by a full neuropsychological battery or self-reported measures of NCI that included z-scores based on published normative data	Self-reported measures of NCI that did not include z-scores based on published normative data Screening instruments of cognitive impairment (e.g. IHDS; HDS; MoCA, etc.)

### 2.2. Search strategies

**Figure 1.**
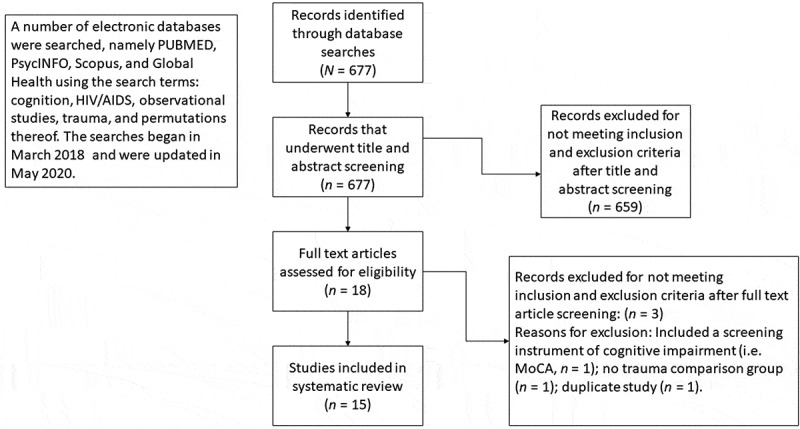
PRISMA flow diagram of search procedure.

Prior to conducting the searches, we collaboratively decided on the search terms. A number of electronic databases were searched, namely PUBMED, PsycINFO, Scopus, and Global Health using the search terms: cognition, HIV/AIDS, observational studies, trauma, and permutations thereof. The searches began in March 2018. A total of 677 abstracts were extracted to a Rayyan database, a web and mobile application for systematic reviews (Ouzzani, Hammady, Fedorowicz, & Elmagarmid, 2016). The searches were updated in May 2020 using the following electronic databases: PUBMED, Psych Info, and Google Scholar. Reference lists of eligible studies were also scanned. The abstracts were then reviewed independently by three reviewers to see if they met inclusion criteria. A face-to-face meeting was subsequently held whereby reviewers discussed discrepancies and reached consensus about which studies to include. Each reviewer flagged duplicate entries on Rayyan, with removal of the duplicates from the database via consensus. See Prisma flow diagram (Figure 1).

### 2.3. Data analysis

Data were extracted by two independent reviewers using a standard data extraction form. The form included the following fields: author and date, country, study design, sample, trauma, and PTSD measurement and summary of NCI measures. Quality appraisal of included studies was conducted by GS and SM. However, given that the seminal studies were conducted by two independent reviewers, a third objective reviewer was brought in to assist with the quality appraisal of these studies. The quality appraisal was guided by the Systematic Appraisal of Quality in Observational Research (SAQOR) tool that comprises six domains (each containing two to five questions): sample, control/comparison group, exposure/outcome measurements, follow-up, confounders, and reporting of data (Ross et al., 2011). Table 2 presents the quality appraisal.

**Table 2.** Quality assessment of papers included in the systematic review (SAQOR tool).

Study	Sample	Country	Control/comparison group	Exposure/outcome measures	Distorting influences	Reporting of data	Overall study quality
Clark et al., 2012	Sample recruited 49 HIV+ and 47- individuals with ELS to explore effects of HIV and ELS on Amygdala morphometry and NCI. In summary, sample is clear.	USA	Comparison group (47 HIV- individuals) included, easily identifiable, source of comparison group clear. In summary, comparison group is clear.	Main exposure: Early Life Stress Questionnaire Main outcome: sMRI and assessments of psychomotor speed, executive functions, verbal memory and fine motor dexterity. In summary, quality of exposure/outcome measurement is adequate	Confounders (demographic variables that differed significantly between groups). In summary, description of influences are unclear.	Missing data Missing data not reported. In summary, reporting of data is unclear. However, data are clearly and accurately presented with p values reported. No confidence intervals reported. In summary, reporting of data is adequate.	Moderate
Clark et al., 2018	Sample recruited 44 HIV+ participants to explore changes in brain volume affected by ELS. Source, method and entry criteria clearly stated. In summary, sample is adequate.	USA	Comparison group included, easily identifiable, source of comparisons explained, comparisons matched, differences between cases and comparisons controlled for. In summary, comparison group is adequate.	Main exposure: Early Life Stress Questionnaire (ELSQ) Main outcome: Reaction Time Task Structural MRI Self-reported cognitive function through The HIV Medical Outcomes Survey (MOS-HIV). In summary, quality of exposure/outcome measurement is adequate.	Confounders (neuropsychiatric symptoms and scanner type) adequately controlled for. In summary, description of influences are adequate.	Missing data not reported. However, data are clearly and accurately presented with p values reported. No confidence intervals reported. In summary, reporting of data is adequate.	High
Deiss et al., 2019	Cohort recruited 200 military beneficiaries living with HIV to explore the relationship between NCI and mental health disorders. Source of the cohort is unclear. Method and entry criteria clearly stated. In summary, cohort is unclear.	USA	Comparison group included (individuals without lifetime history of PTSD), easily identifiable, source of comparison group unclear. In summary, comparison group is unclear.	Main exposure: Composite International Diagnostic Interview. (CIDI) Main outcome: NCI battery measuring verbal fluency, attention/working memory, visuospatial functioning, information processing, learning/recall, executive functions, motor speed and dexterity and effort.). In summary, quality of exposure/outcome measurement is adequate.	Confounders (age, ethnicity, education) adequately controlled for. Potential confounders including cumulative traumas, war deployed to and region of residence not described. In summary, description of influences are unclear.	Missing data not reported. However, data are clearly and accurately presented with p values and confidence intervals reported. In summary, reporting of data is adequate.	Low
Kapetanovic et al., 2020	187 individuals (102 with HIV and IPT; 35 with HIV only; 24 HIV-negative with IPT; 26 HIV-negative). Source of the cohort is clear. Method and entry criteria clearly stated. In summary, sample is adequate.	USA	Comparison group included (HIV-positive with no IPT and HIV-negative with and without IPT), easily identifiable, source of comparisons explained, comparisons matched, differences between cases and comparisons controlled for. In summary, comparison group is adequate.	Main exposure: Client Diagnostic Questionnaire (CDQ). Main outcome: Structural MRI and assessments of attention/working memory, psychomotor speed, executive functions, information processing, verbal fluency, learning, memory, and adult reading In summary, quality of exposure/outcome measurement is adequate.	Confounders (group status, sex, age, race, and education) were adjusted for. Already adjusted T-scores were applied. Models were also adjusted for WTAR (adult reading) scores. In summary, description of influences are clear.	Data are clearly and accurately presented with p values and confidence intervals reported. In summary, reporting of data is adequate.	High
Lin et al., 2011	964 HIV+ individuals with TBI. Source, method and entry criteria clearly stated. In summary, sample is adequate.	USA	Comparison group included (individuals without TBI), easily identifiable, source of comparison group clear. In summary, comparison group is clear.	Main exposure: self-reported medical history. Main outcome: NCI battery measuring overall vocabulary, verbal fluency, attention/working memory, learning, information processing, learning/recall, executive functions, motor speed and dexterity). In summary, quality of exposure/outcome measurement is adequate.	Confounders (age, sex, education, and ethnicity) adequately controlled for. However, number of TBIs and type of TBI were not controlled for. In summary, description of influences are adequate.	Missing data adequately reported.	Moderate
Malan-Muller et al., 2013	The sample recruited 128 women.	South Africa	The sample included *n* = 83 who were HIV positive.	Main exposure: childhood trauma measured by the childhood trauma questionnaire (CTQ) measuring 28 experiences of childhood trauma. Main outcome: neurocognitive testing using the International Neuropsychological Test Battery developed by the HIV Neurobehavioral Research Centre (HNRC). A research psychologist conducted the testing examining motor, verbal fluency, attention, working memory, speed, learning recall and executive function.	Confounders (age, education, Body Mass Index, trauma sub-type, traumatic life experiences, post-traumatic stress disorder (PTSD), symptomatology and alcohol abuse), LTL, ARV treatment. As all participants were black so ethnicity was not controlled for in the analysis.	Missing data not mentioned.	High
Spies et al., 2012	Sample recruited consisted of 83 (64%) HIV positive and 47 (36%) HIV-negative women.	South Africa	Forty-eight HIV-positive women were exposed to childhood trauma. Among controls, 20 had been exposed to childhood trauma.	Main exposure: childhood trauma measured by the childhood trauma questionnaire (CTQ) measuring 28 experiences of childhood trauma. Main outcome: neurocognitive testing using the International Neuropsychological Test Battery developed by the HIV Neurobehavioral Research Centre (HNRC). A research psychologist conducted the testing examining motor, verbal fluency, attention, working memory, speed, learning recall and executive function.	Age and education corrected z-scores were calculated from all raw NP data and grouped into domains (motor, verbal fluency, working memory, speed, learning, recall, and executive functions).	Missing data not reported. Both statistically and non-statistically significant variables are reported.	High
Spies et al., 2016	A sample of 124 women tested for HIV status were recruited.	South Africa	62 were HIV-positive, 32 with a history of childhood trauma and 30 without, and 62 were HIV-negative, 31 with a history of childhood trauma and 31 without.	Main exposure: childhood trauma measured by the childhood trauma questionnaire (CTQ) measuring 28 experiences of childhood trauma. Main outcome: Neurocognitive function was measured with 10 tests that have been used commonly in HIV research and cover seven ability domains (learning, delayed recall, processing speed, attention/working memory, executive function, verbal fluency, motor ability).	Age, marital status, ethnicity, employment status, years of education and estimated intracranial vault, brain volumes were captured. Estimated intracranial vault was added as a covariate to control for individual differences in head size.	Missing data not reported. Both statistically and non-statistically significant variables are reported.	High
Spies et al., 2017	A sample of *n* = 67 (51.9%) HIV+ and 50(38.8%) HIV− women.	South Africa	Fifty-three HIV+ women and 18 controls were exposed to childhood trauma.	Main exposure: childhood trauma measured by the childhood trauma questionnaire (CTQ) measuring 28 experiences of childhood trauma. Main outcome: Neurocognitive function was measured with 10 tests that have been used commonly in HIV research and cover seven ability domains (learning, delayed recall, processing speed, attention/working memory, executive function, verbal fluency, motor ability)	Age, marital status, ethnicity, years of education and employment status were captured. A general physical examination was conducted on all participants. Virologic markers of disease progression (CD4 lymphocyte count and viral loads) were captured.	Missing data not reported.	High
Pukay-Martin et al., 2003	251 HIV+ and 82 HIV- gay or bisexual men. Source clearly stated. Method and entry criteria unclear. In summary, sample is unclear.	USA	Comparison group included (HIV- men), easily identifiable, source of comparison group clear. In summary, comparison group is clear.	Main exposure: Psychiatric Epidemiology Research Interview (PERI) Life Events Scale, measuring 47 traumatic events. Main outcome: Wechsler Adult Intelligence Scale-Revised, Selective Reminding Test, Visual. Memory Span Forward and Backward from the Wechsler Memory Scale-Revised, Verbal Concept Attainment Test, Wisconsin Card Sorting Test, Verbal Fluency, Figural Fluency, Trail Making A and B, Grooved Pegboard, and the Paced Auditory Serial Addition Test. In summary, quality of exposure/outcome measurement is adequate.	Confounders (impact of anxiety, depression, age and education) controlled for. In summary, description of influences are adequate.	Missing data and data analysis procedures not reported. However, data are clearly and accurately presented with p values reported. No confidence intervals reported. In summary, reporting of data is adequate.	High
Rubin et al., 2015	Sample recruited 1009 HIV-infected and 496 at-risk HIV-uninfected women to explore the association between perceived stress and verbal memory from the Women’s Inter-Agency HIV Study (WIHS). In summary, sample is clear.	USA	Comparison group included 496 at-risk HIV-uninfected women, easily identifiable, source of comparison group clear. In summary, comparison group is clear.	Main exposure: Perceived Stress Scale (PSS-10) Main outcome: verbal memory performance using the Hopkins Verbal Learning Test (HVLT). Other domains assessed included verbal learning, attention and concentration, executive functioning (behavioural inhibition, mental flexibility, working memory), psychomotor speed, verbal fluency, and fine motor skills. In summary, quality of exposure/outcome measurement is adequate. In summary, quality of exposure/outcome measurement is adequate.	Confounders (annual household income; depression; Hepatitis C status; self- reported recent, former, or never use of marijuana, crack, cocaine, and/or heroin and smoking; self-reported recent heavy alcohol use; recent antidepressant use; and study geographic site. Additional HIV-related clinical variables of interest included cART use and self-reported adherence, duration of antiretroviral therapy use, current CD4 count <200 cells/mm3, current viral load (undetectable, <10,000 cp/ml, ≥10,000 cp/ ml), and nadir CD4 count <200 cells/mm3 adequately reported. In summary, description of influences are adequate.	Missing data adequately reported. However, data are clearly and accurately presented with p values reported. No confidence intervals reported. In summary, reporting of data is adequate.	High
Rubin et al., 2016	Sample recruited included 38 HIV+ women from the Women’s Inter-Agency HIV Study (WIHS) to explore the neurobiological factors contributing to stress-related memory impairment. In summary, sample is clear.	USA	Comparison group (HIV-infected low stress) included, easily identifiable, source of comparison group clear. In summary, comparison group is clear.	Main exposure: Perceived Stress Scale (PSS-10) and PTSD Checklist-Civilian Version (PCL-C) Main outcome: The Hopkins Verbal learning Test (HVLT) to measure verbal learning and memory. Additional cognitive domain measured included: attention/concentration, executive functions, psychomotor speed and verbal fluency sMRI to measure brain volumetric abnormalities In summary, quality of exposure/outcome measurement is adequate.	Confounders (age) adequately reported. In summary, description of influences are adequate.	Missing data not reported. In summary, reporting of data is unclear. However, data are clearly and accurately presented with p values reported. No confidence intervals reported. In summary, reporting of data is adequate.	High
Rubin et al., 2017	Sample recruited included 646 HIV+ and 300 HIV- women from the Women’s Inter-Agency HIV Study to explore the association between perceived stress and PTSD in learning, memory, and fluency impairments. In summary, sample is clear.	USA	Comparison group (300 HIV- women) included, easily identifiable, source of comparison group clear. In summary, comparison group is clear.	Main exposure: Perceived Stress Scale (PSS-10) and PTSD Checklist-Civilian version (PCL-C) Main outcome: learning and memory. Other domains measure included attention, executive function, fluency, psychomotor speed, and motor skills In summary, quality of exposure/outcome measurement is adequate.	Confounders (wave of enrolment, self-reported annual household income, harmful alcohol use, smoking status, marijuana use, and crack, cocaine, and/or heroin use, psychiatric antidepressant/antipsychotic medication, and positive Hepatitis C antibody) adequately reported. In summary, description of influences are adequate.	Missing data not reported. In summary, reporting of data is unclear. However, data are clearly and accurately presented with p values reported. No confidence intervals reported. In summary, reporting of data is adequate.	High
Watson et al., 2018	Sample recruited included 122 HIV+ and 95 HIV- individuals from the Multi-Dimensional Successful Ageing among Adults living with HIV study to explore the effects of trauma, economic hardship, and stress on NCI and everyday function. In summary, sample is clear.	USA	Comparison group (95 HIV- individuals) included, easily identifiable, source of comparison group clear. In summary, comparison group is clear.	Main exposure: self-report Women’s Health Initiative (WHI) Life Events Scale Main outcome: executive functioning, learning, memory (delayed recall), working memory, verbal fluency, speed of information processing, and complex motor skills. In summary, quality of exposure/outcome measurement is adequate.	Confounders (gender, years of education, lifetime MDD, lifetime substance use disorder, lifetime alcohol use, race/ethnicity and lifetime cannabis use) adequately reported. In summary, description of influences are adequate.	Missing data not reported. In summary, reporting of data is unclear. However, data are clearly and accurately presented with p values reported. No confidence intervals reported. In summary, reporting of data is adequate.	High
Womersley et al., 2018	Sample recruited 128 HIV positive women between 18 and 65 years of age, who were fluent in isiXhosa, English and/or Afrikaans and with a minimum literacy level of 5^th^ grade education.	South Africa	The study examines how childhood trauma interacts with ApoE in relation to NCI so comparison is determined by childhood trauma versus no childhood trauma.	Main exposure: childhood trauma measured by the childhood trauma questionnaire (CTQ) measuring 28 experiences of childhood trauma. Main outcome: Neurocognitive function as measured by the Neurobehavioral Research Centre (HNRC) battery. This battery consists of 17 tests and uses culturally adapted for the South African context. Seven domains of cognitive function: motor ability, processing speed, verbal fluency, learning, delayed recall, attention, working memory and executive function were examined.	Confounder included global cognitive scores.	Missing data not reported. However, data are clearly and accurately presented with p values reported.	High

SAQOR – Systematic Appraisal of Quality in Observational Research.

## 3. Results

### 3.1. Quality assessment of included studies

The majority of studies (*n* = 12; 80%) included were deemed to be of high quality. Two studies were of moderate quality and only one study was deemed to be of low quality due to inadequate description of distorting influences/confounders and the sample and comparison groups. See Table 2 for more detail on quality appraisal of included studies.

### 3.2. Study characteristics

Tables 3 and 4 provide characteristics of the fifteen selected studies meeting inclusion criteria. No studies including a mixture of eligible and non-eligible participants (e.g. both adults and children) were found; therefore, data disaggregation was not necessary. Collectively the fifteen studies included 5140 participants (3866 PLWHA), with the average age of participants in most studies in the 30s and 40s. The average age for fourteen of fifteen studies, (one study reported the age range only) was 40 years. Eight were all-female studies (Malan-Muller et al., 2013; Rubin et al., 2017, 2015, 2016; Spies et al., 2016, 2012; Spies, Fennema-Notestine, Cherner, & Seedat, 2017; Womersley, Spies, Seedat, & Hemmings, 2019) and two were all-male studies (Deiss et al., 2019; Pukay-Martin, Cristiani, Saveanu, & Bornstein, 2003), with the remainder (five studies) including both men and women (Clark, Arce Renteria, Hegde, & Morgello, 2018; Clark et al., 2012; Kapetanovic et al., 2020; Lin et al., 2011; Watson et al., 2019).

**Table 3.** Studies included in the comprehensive review of the literature on the association between potentially traumatic or stressful events and cognitive function among PLWHA.

Study	Total	PLWHA *n*	HIV *n*	HIV+ comparison group	Country	Male (%)	Age range or mean	Trauma or stress measure	Mental health assessment	Relationship between trauma and NCI in PLWHA
Clark et al., 2012	96	49	47	-	USA	58	45	ELSQ	-	**
Clark et al., 2018	44	44	-	Yes	USA	59	45	ELSQ	-	*
Deiss et al., 2019	189	189	-	Yes	USA	100	28–44	-	CIDI (current and lifetime history of alcohol use disorder, depression, and PTSD)	**
Kapetanovic et al., 2020	187	137	50	Yes	USA	63	50	CDQ trauma inventory	CDQ	*
Lin et al., 2011	964	964	-	Yes	USA	77	44	Self-reported medical history	-	*
Malan-Muller et al., 2013		83	45	-	South Africa	0	30	CTQ-SF	-	**
Spies et al., 2012	128	83	47	-	South Africa	0	30	CTQ-SF	-	*
Spies et al., 2016	124	62	62	-	South Africa	0	30; 18–50	CTQ-SF	-	*
Spies et al., 2017	117	67	50	-	South Africa	0	33; 18–50	CTQ-SF	-	**
Pukay-Martin et al., 2003	333	251	82	-	USA	100	33	PERI Life Events Scale	-	**
Rubin et al., 2015	1499	1003	496	-	USA	0	46; 25–87	-	PSS-10	***
Rubin et al., 2016	38	38	-	Yes	USA	0	44; 27–59	PCL-C	PSS-10	*
Rubin et al., 2017	964	646	300	-	USA	0	45	PCL-C	PSS-10	*
Watson et al., 2019	217	122	95	-	USA	78	51; 35–65	WHI Life Events Scale	-	*
Womersley et al., 2019	128	128	-	Yes	South Africa	0	33; 21–50	CTQ-SF		*

* = *p* < 0.05; ** = *p* < 0.01; *** = *p* < 0.001

CDQ = Client Diagnostic Questionnaire; CIDI = Composite International Diagnostic Interview; CTQ-SF = Childhood Trauma Questionnaire-Short Form; ELSQ = Early Life Stress Questionnaire; PCL-C = PTSD Checklist-Civilian Version; PERI = Psychiatric Epidemiology Research Interview; PSS-10 = Perceived Stress Scale; WHI = Women’s Health Initiative Life Events Scale.

**Table 4.** HIV disease parameters of included studies.

Study	PLWHA *n*	% on ARVs	Mean (SD) duration of illness in years		Mean (SD) CD4 T-cell count (cells/mm^3)^		Mean (SD) HIV viral load (copies/mL)	
Clark et al., 2012	49	84	High ELS (*n* = 25) 13.04 (7.98)	Low ELS (*n* = 24) 14.46 (6.60)	High ELS (*n* = 25) 460.92 (257.35)	Low ELS (*n* = 24) 560.96 (313.51)	High ELS (*n* = 25) 20 Undetectable (<75) 4 High (>400)	Low ELS (*n* = 24) 16 Undetectable (<75) 6 High (>400)
Clark et al., 2018	44	100	High ELS (*n* = 26) 16.00 (7.22)	Low ELS (*n* = 18) 14.56 (6.64)	High ELS 608.85 (272.19)	Low ELS 594.28 (298.40)	High ELS 65% <50	Low ELS 53% <50
Deiss et al., 2019	189	66	5 (2.11 median IQR)		542 (413,697 median IQR)		48 copies/mL (48,3282 median IQR)	
Kapetanovic et al., 2020	137	100	IPT+ (*n* = 102) 17.9 (9.0)	IPT- (*n* = 35) 14.1 (9.7)	IPT+ (*n* = 102) Nadir CD4 201.5 (177.5)	IPT- (*n* = 35) Nadir CD4 217.9 (169.1)	IPT+ (*n* = 102) All were aviremic (<40 copies/mL)	IPT- (*n* = 35) All were aviremic (<40 copies/mL)
Lin et al., 2011	964	66	nr		TBI+ (*n* = 110) Nadir CD4 213.3 (188.5)	TBI- (*n* = 590) Nadir CD4 219.9 (210.7)	TBI+ (*n* = 110) 65.1% detectable (based on limit of detection of 50)	TBI- (*n* = 590) 70.1% detectable (based on limit of detection of 50)
Malan-Muller et al., 2013	83	16	nr		nr		nr	
Spies et al., 2012	83	16	nr		405 (259.80)		105,169.5 (407,459.5)	
Spies et al., 2016	62	49	nr		431.20 (267.75)		16,172.08 (439,037.76)	
Spies et al., 2017	67	81	nr		CT+ (*n* = 53) 442.04 (234.88)	CT- (*n* = 14) 413.57 (285.02)	CT+ (*n* = 53) 61,505.04 (174,046.09)	CT- (*n* = 14) 81,169.93 (185,206.44)
Pukay-Martin et al., 2003	251	nr	nr		49.1% ˃400mm^3^; 27.5% ≥ 200mm^3^ and ≤ 400 mm^3^; 23.4% ˂200mm^3^		nr	
Rubin et al., 2015	1003	76	nr		High stress (*n* = 384) Nadir 208 (157) 44% ˃500 40% ≥200 and <500 16% <200	Low stress (*n* = 619) Nadir 218 (158) 55% ˃500 34% ≥200 and <500 11% <200	High stress (*n* = 384) 47% undetectable 37% <10,000 16% ≥10,000	Low stress (*n* = 619) 55% undetectable 32% <10,000 13% ≥10,000
Rubin et al., 2016	38	79	nr		High stress (*n* = 18) Nadir 287 (177) 28% ˃500 55% ≥200 and <500 17% <200	Low stress (*n* = 20) Nadir 325 (226) 74% ˃500 16% ≥200 and <500 10% <200	High stress (*n* = 18) 44% undetectable 28% <10,000 28% ≥10,000	Low stress (*n* = 20) 70% undetectable 20% <10,000 10% ≥10,000
Rubin et al., 2017	646	77	nr		521 (401 median IQR)		29% <500	
Watson et al., 2018	122	96	17.1 (8.7)		633(425, 851 median IQR)		6.6% detectable	
Womersley et al., 2018	128	46	nr		nr		nr	

nr = not reported; ELS = early life stress, TBI = traumatic brain injury; CT = childhood trauma; IQR = interquartile range

#### 3.2.1. Study design

There were ten case-control studies (Clark et al., 2012; Kapetanovic et al., 2020; Malan-Muller et al., 2013; Pukay-Martin et al., 2003; Rubin et al., 2017, 2015; Spies et al., 2016, 2012, 2017; Watson et al., 2019) and five cross-sectional studies (Clark et al., 2018; Deiss et al., 2019; Lin et al., 2011; Rubin et al., 2016; Womersley et al., 2019). Eight studies were longitudinal in design (Malan-Muller et al., 2013; Rubin et al., 2017, 2015, 2016; Spies et al., 2016, 2012, 2017; Womersley et al., 2019) but only one of these presented longitudinal data examining the relationship between childhood trauma and HIV and change in cognition over time (Spies et al., 2017) and one examined the relationship between perceived stress and PTSD and NCI over time (Rubin et al., 2017).

#### 3.2.2. Study location

All fifteen studies were limited to two countries; ten studies (67%) were from the USA (high-income country) and five (33%) from South Africa (middle-income country). No studies from low-income countries/regions met entry criteria.

#### 3.2.3. Trauma measurement

The majority of studies (*n* = 12) measured trauma using self-report questionnaires, with the exception of one study which included self-reported medical histories of Traumatic Brain Injury (TBI) to examine if PLWHA with a clear history of TBI had greater risk of NCI compared to those without TBI (Lin et al., 2011), one study which included the Composite International Diagnostic Interview (CIDI) to assess for a DSM-IV diagnosis of PTSD and other mental health conditions (Deiss et al., 2019), and another study which included the Client Diagnostic Questionnaire (CDQ) to assess for interpersonal trauma (IPT history) and PTSD. Overall, current or past PTSD was assessed for in four studies (Deiss et al., 2019; Kapetanovic et al., 2020; Rubin et al., 2017, 2016).

#### 3.2.4. Trauma exposure

Of the fifteen studies, seven studies (Clark et al., 2018, 2012; Malan-Muller et al., 2013; Spies et al., 2016, 2012, 2017; Womersley et al., 2019) examined early life trauma only (experienced before the age of 18) and seven studies (Deiss et al., 2019; Lin et al., 2011; Pukay-Martin et al., 2003; Rubin et al., 2017, 2015, 2016; Watson et al., 2019) examined adult-onset trauma only (experienced after age 18). Only one study considered both early life and adult-onset trauma (Kapetanovic et al., 2020) but no studies sought to separate out the effects of early-onset versus adult-onset trauma. One study from the USA assessed the combined effects of PTEs (through a perceived stress measure) and PTSD (PTEs + PTSD) on NCI (Rubin et al., 2017). Across the studies, sample sizes ranged from 38 to 1003 PLWHA. In ten of the fifteen studies (67%) a HIV-negative control group was included, with HIV-negative control groups ranging in size from 45 to 496. The remaining five studies did not include a HIV-negative control group but included a comparison group comprised of PLWHA.

### 3.3. Findings on trauma and NCI

All fifteen included studies that assessed NCI in PLWHA demonstrated a significant association with trauma exposure (Table 5). Five studies controlled for additional confounding variables such as depression, substance use, and antidepressant use (Clark et al., 2018, 2012; Pukay-Martin et al., 2003; Rubin et al., 2015; Watson et al., 2019). Among the fifteen studies measuring both trauma and NCI in PLWHA, three studies (conducted in the USA) also estimated the prevalence of NCI (Deiss et al., 2019; Kapetanovic et al., 2020; Watson et al., 2019). The prevalence of NCI in these studies was 19% (Deiss et al., 2019), 39% (Watson et al., 2019), and 27% (Kapetanovic et al., 2020) respectively. One study that recruited participants from the CNS HIV Antiretroviral Therapy Effects Research (CHARTER) estimated NCI using a global deficit score (Lin et al., 2011). The remaining studies reported associations between exposure to PTEs and specific cognitive domains (Table 5).

**Table 5.** Relationship between potentially traumatic or stressful life events and cognitive function among PLWHA.

			Cognitive domains						
Study	PLWHA *n*	On ARVs (%)	EF	FLU	ATT/WM	PS	LRN/MEM	Motor	Global NCI
Clark et al., 2012	49	84				**			
Clark et al., 2018	44	100			*				
Deiss et al., 2019	189	66							**
Kapetanovic et al., 2020	137	100			*				
Lin et al., 2011	964	66	*		*				
Malan-Muller et al., 2013	83	16		**					
Spies et al., 2012	83	16				*	*		
Spies et al., 2016	62	49	*	*	*	*	*	*	
Spies et al., 2017	67	81	**	**					
Pukay-Martin et al., 2003	251	nr	*	**	**	*		*	**
Rubin et al., 2015	1003	76					***		
Rubin et al., 2016	38	79					*		
Rubin et al., 2017	646	77		*			**		
Watson et al., 2019	122	96	*		*		*		
Womersley et al., 2019	128	46			*			*	

nr = not reported; * = *p* < 0.05; ** = *p* < 0.01; *** = *p* < 0.001

Cognitive domains: EF = executive function; FLU = Fluency; ATT/WM = attention and working memory; PS = processing speed; LRN/MEM = learning and memory; VS = visuospatial and visuoconstructive functions; NCI = neurocognitive impairment.

#### 3.3.1. Cognitive domains

In these studies, a cognitive domain was assessed using more than one test. Five studies demonstrated an association between PTEs and SLEs and executive function deficits (Lin et al., 2011; Pukay-Martin et al., 2003; Spies et al., 2016, 2017; Watson et al., 2019). Regarding verbal fluency, five studies demonstrated an association with PTEs and SLEs (Malan-Muller et al., 2013; Pukay-Martin et al., 2003; Rubin et al., 2017; Spies et al., 2016, 2017; Watson et al., 2019). For attention/working memory, seven studies demonstrated an association with trauma (Clark et al., 2018; Kapetanovic et al., 2020; Lin et al., 2011; Pukay-Martin et al., 2003; Spies et al., 2016; Watson et al., 2019; Womersley et al., 2019); four studies demonstrated an association with processing speed, (Clark et al., 2012; Pukay-Martin et al., 2003; Spies et al., 2016, 2012); six studies demonstrated an association with learning (Rubin et al., 2017, 2015, 2016; Spies et al., 2016, 2012; Watson et al., 2019) while three studies demonstrated an association with motor function (Pukay-Martin et al., 2003; Spies et al., 2016; Womersley et al., 2019). Two studies (Deiss et al., 2019; Pukay-Martin et al., 2003) reported an association between global NCI and trauma (i.e. not distinguished by cognitive domain).

#### 3.3.2. Associations between PTEs and SLEs and NCI

Across all fifteen studies, there was consistent evidence for an association between PTEs and SLEs and NCI. In the nine studies that included a HIV-negative control group, results consistently showed that trauma was a significant risk factor for neurocognitive dysfunction among PLWHA. For example, a recent study showed that interpersonal trauma (IPT) exposure and HIV infection have a synergistic effect on daily functioning and cortical thickness in PLWHA (Kapetanovic et al., 2020). Participants were classified as IPT+ if they experienced any of the following: childhood physical or sexual abuse, intimate partner violence as an adult, physical, or sexual assault as an adult, direct combat, seeing people harming one another in the family as a child, or losing a child to death (Kapetanovic et al., 2020). Of all fifteen studies included, this was the only study that considered both early life and adult-onset trauma, although this study did not separate out the effects of early-onset versus adult-onset trauma on NCI in PLWHA. This study found that attention/working memory test performances were significantly worse in PLWHA with IPT compared with PLWHA without IPT and HIV-negative counterparts without IPT. The HIV+ IPT+ group had significantly greater compromise in activities of daily living and also had ‘impaired’ scores on a measure of subjective cognitive function compared with the remaining three groups (Kapetanovic et al., 2020). In another study, a case control design, stressful life events (47 events in seven different categories: relationships and love, family, residence, crime and legal matters, finances, health, and other), measured by the Psychiatric Epidemiology Research Interview (PERI) Life Events Scale, were associated with cognitive impairment only among male PLWHA (Pukay-Martin et al., 2003). In a cohort study, where participants were recruited across six USA based sites, a significant HIV by stress interaction was found for verbal memory among HIV-infected women only, with high stress associated with lower performance on verbal memory tests compared to women with low stress (Rubin et al., 2015). In another cohort study, assessing the combined effects of multiple traumatic and stressful experiences among PLWHA, a higher composite stress score (consisting of multiple adverse experiences including trauma, economic hardship, and stress) was associated with poorer executive function, learning, working memory, and greater decline in activities of daily living, even after controlling for relevant demographic, psychiatric, substance use, and HIV disease covariates. On their own, individual stress composite scores did not predict these outcomes, suggesting that the accumulation of adverse experiences may additively or synergistically harm cognitive health (Watson et al., 2019). In the remaining five studies, that included a comparator group of PLWHA, similar results were demonstrated. In two studies, one a USA based cross-sectional study design and the other a USA based MRI study, HIV-positive participants were categorised into low stress and high stress groups (Clark et al., 2018; Rubin et al., 2016). In one of these studies by Rubin et al., which compared HIV-infected women with low stress PSS-10 scores in the lower two tertiles) and HIV-infected women with high stress (PSS-10 scores in the top tertile), high stress and HIV seemed to be associated with worse performance on measures of verbal learning and memory and smaller brain volumes (Rubin et al., 2016). Similarly in the other study by Clark et al, participants with high early life stress (ELS) (defined as an endorsement of three or more adverse childhood events [ACEs]) exhibited poorer working memory compared to those with low-ELS (defined by endorsement of fewer than three ACEs) (Clark et al., 2018). In studies that included comparison groups with and without trauma (viz. childhood trauma) (Womersley et al., 2019), PTSD (Deiss et al., 2019), and TBI (Lin et al., 2011), PLWHA who were trauma exposed consistently performed more poorly than their counterparts without trauma.

#### 3.3.3. PTSD findings

Overall, current or past PTSD was assessed for in four studies (Deiss et al., 2019; Kapetanovic et al., 2020; Rubin et al., 2017, 2016). PTSD was not dealt with as a mediator of trauma exposure and NCI but rather as an independent predictor of NCI in one study (Deiss et al., 2019). Although PLWHA were assessed for PTSD, the independent effect of PTSD on NCI was not reported in two of these studies (Kapetanovic et al., 2020; Rubin et al., 2016). Instead, the effects of high vs. low stress (viz. perceived stress) (Rubin et al., 2016) and interpersonal trauma (IPT) (Kapetanovic et al., 2020) on NCI were reported. In the study including PTSD as an independent predictor of NCI, lifetime history of PTSD was independently associated with NCI in a cohort of 189 U.S. military men living with HIV (Deiss et al., 2019). Individuals with a lifetime history of PTSD were six times more likely to be diagnosed with NCI than individuals without PTSD (Deiss et al., 2019).

#### 3.3.4. PTE exposure and PTSD findings

The fourth study reporting on current or past PTSD sought to assess both the separate and interactive effects of HIV and stress or PTSD on NCI (Rubin et al., 2017). This longitudinal study from the USA assessed the combined effects of PTEs (through a perceived stress measure) and PTSD on NCI (Grant, 2008), finding that higher levels of stress (viz. perceived stress) and PTSD symptoms, more so than depressive symptoms, may contribute to different patterns of detrimental neurocognitive performance among PLWHA, compared to lower levels of stress and PTSD (Rubin et al., 2017). Across time points, PTSD was associated with lower performance on both learning and memory among women living with HIV but not HIV-negative counterparts. This longitudinal study provides evidence that elevated perceived stress and PTSD symptoms in the context of HIV are linked to alterations in verbal abilities (learning, memory, fluency) over time (Rubin et al., 2017).

## 4. Discussion

### 4.1. Associations between PTEs and SLEs and NCI

In this paper we present, to our knowledge, the most comprehensive review of evidence of the relationship between broad concepts of PTEs and NCI in PLWHA. Despite the small evidence base (especially from low and middle-income countries [LMIC]) and the methodological limitations of the available studies, it is notable that in all the studies included there was an association between trauma and NCI in PLWHA. In order to provide a robust body of evidence, the studies in the present review include measures of different kinds of adversity including exposure to PTEs (both early life and adult-onset trauma), economic hardship (food insecurity and low SES), perceived stress, and traumatic brain injury. These findings of the relationship between NCI and PTE have emerged in several previous studies (Pavlovic, Pekic, Stojanovic, & Popovic, 2019). Perceived stress may include stressful life events considered as potentially traumatic events. Stressful life events are thought to increase risk for disease when one perceives that the demands these events impose tax or exceed a person’s adaptive capacity (Cohen, Janicki-Deverts, & Miller, 2007). Exposures to chronic stress are considered the most toxic because they are most likely to result in long-term or permanent changes in the emotional, physiological, and behavioural responses that influence susceptibility to and course of disease (McEwen, 2006). A high level of perceived stress has been shown to be accompanied by symptoms of depression and/or anxiety (Wiegner, Hange, Björkelund, & Ahlborg, 2015). Irrespective of the type of exposure, the results show compelling evidence for the contributory role of trauma in NCI in PLWHA. All 15 included studies that assessed NCI in PLWHA demonstrated a significant association with PTEs and SLE and/or PTSD as broadly defined in our study. Three studies reported prevalences of 19%, 39%, and 27% of NCI (Deiss et al., 2019; Kapetanovic et al., 2020; Watson et al., 2019). All 15 included studies demonstrated an association of PTEs and SLEs and/or PTSD and various cognitive domains, including executive function (Lin et al., 2011; Pukay-Martin et al., 2003; Spies et al., 2016, 2017; Watson et al., 2019), attention/working memory (Clark et al., 2018; Kapetanovic et al., 2020; Lin et al., 2011; Pukay-Martin et al., 2003; Spies et al., 2016; Watson et al., 2019; Womersley et al., 2019), processing speed (Clark et al., 2012; Pukay-Martin et al., 2003; Spies et al., 2016, 2012), verbal fluency (Malan-Muller et al., 2013; Pukay-Martin et al., 2003; Rubin et al., 2017; Spies et al., 2016, 2017), learning/memory (Rubin et al., 2017, 2015, 2016; Spies et al., 2016, 2012; Watson et al., 2019), and motor function (Pukay-Martin et al., 2003; Spies et al., 2016; Womersley et al., 2019), with some studies showing an association with global cognitive function among PLWHA (Deiss et al., 2019; Pukay-Martin et al., 2003).

### 4.2. Methodological considerations of included studies

Potential confounders were adequately controlled for in the majority of studies including (Clark et al., 2018; Deiss et al., 2019; Lin et al., 2011; Malan-Muller et al., 2013; Pukay-Martin et al., 2003; Rubin et al., 2017, 2015, 2016; Spies et al., 2016, 2012; Watson et al., 2019). A recent systematic review by Rubin and Maki found that depression contributed to impairments particularly in the domains of executive function, processing speed, learning, and motor function (Rubin & Maki, 2019b), highlighting the importance for studies to adequately consider the effects of depression on NCI or control for these effects. In some studies included in this review, depression was controlled for or included in analyses to determine the independent effects thereof on NCI (Clark et al., 2018, 2012; Deiss et al., 2019; Lin et al., 2011; Pukay-Martin et al., 2003; Rubin et al., 2017, 2015). The majority of studies were cross-sectional and as such, a causal relationship between trauma and NCI cannot be inferred. However, in the longitudinal study by Spies et al., the effects of HIV and childhood trauma on NCI were sustained at 12-month follow-up despite better ART uptake and improved HIV disease status (Spies et al., 2017). In the era of effective ART and increased virologic suppression, the experience of trauma over the life course, the occurrence of stressors, or PTSD among individuals living with HIV infection may hold more relevance in the development of NCI than was previously recognised. Future longitudinal studies are needed. Across studies there were discrepancies in the way trauma, stressors and adversity were defined and measured. Few HIV-trauma-NCI studies included mental health measures (e.g. PTSD, depression etc.) and those that did mostly included self-report screeners of PTSD and depression. Only one study included a structured diagnostic interview to assess PTSD and depression (Deiss et al., 2019). The majority of studies either assessed early-onset or adult-onset trauma. Only one study considered both (Kapetanovic et al., 2020) but no studies sought to separate out the effects of early-onset versus adult-onset trauma. Across studies there were also discrepancies in the measurement of the severity of exposures and in the timing of trauma in relation to NCI. There was a lack of consistency in instrumentation used, both for the assessment of trauma exposure and NCI. Moreover, only three studies estimated the prevalence of NCI based on available norms (Deiss et al., 2019; Kapetanovic et al., 2020; Watson et al., 2019), overwhelmingly studies reported associations between trauma and NCI in the absence of neuropsychological norms (Malan-Muller et al., 2013; Spies et al., 2016, 2012, 2017; Womersley et al., 2019). Across studies, there were inconsistencies in how HIV-related clinical characteristics such as HIV RNA, use of ART, illness stage etc. were dealt with. Moreover, the characterisation of participants across studies in terms of parameters such as gender, age, and HIV co-morbidities was varied. To our knowledge, there are no studies from Europe, South America, or Asia, highlighting the lack of geographical diversity. Finally, more studies are needed to parse out the effects of HIV and PTEs and SLEs on NCI from the effects of HIV and mental health disorders (e.g. PTSD) on NCI.

### 4.3. Clinical implications

The experience of trauma over the life course, the occurrence of highly stressful events, and PTSD deserve more scrutiny in the clinical assessment and management of PLWHA as they may signal the presence of NCI, more than was previously recognised. The findings of the present review, therefore, highlight the need for PTE and SLE screening and detection and implementation of secondary prevention strategies in PLWHA who have PTEs and SLEs. PTEs and SLEs and PTSD symptoms are treatable targets and early intervention may serve to improve cognitive abilities in PLWHA. Trauma-focused interventions in youth and adults, that include resilience building and coping strengthening elements, may be beneficial in mitigating the negative psychological sequelae of PTEs and SLEs. The integration of trauma-focused interventions in primary HIV care aimed at improving cognitive and HIV disease outcomes, as well as emotional well-being and quality of life, among PLWHA, warrants investigation.

### 4.4. Implications for research

The quality analysis of included studies in this review highlights the need for future studies to report their methodology and findings in a standardised way to ultimately improve methodological rigour in observational epidemiological studies. Future research should aim to unpack the mechanisms underlying the association between trauma and NCI among PLWHA. Prospective studies in a range of LMIC and controlling for different types of trauma are needed. Measurement of the severity of exposure and the timing of trauma in relation to NCI should be considered. Due to the cross-sectional nature of these studies, it is not possible to determine the temporal pattern of these changes. Future longitudinal studies that track the course of neurocognitive changes are needed to provide more clarity. Such studies have the potential to provide greater insights into potential targets for therapeutic interventions.

## 5. Limitations

### 5.1. Limitations of reviewed studies

First, the use of self-report screeners of PTSD and other psychiatric diagnoses rather than a structured diagnostic interview in the majority of studies may overestimate the number of individuals meeting criteria for a clinical diagnosis. Second, the heterogeneity of trauma measures and variations in sample size, sampling strategy, and study design, all of which may have impacted on the results reported herein. Third, publication bias, namely a tendency for journals to publish positive findings, may overestimate the strength or consistency of the association between trauma and NCI.

### 5.2. Limitations of this study

In our study, trauma was broadly defined as potentially traumatic events (PTEs) and stressful live events (SLEs) during childhood and/or adulthood. Given the paucity of studies in this area, both studies of potentially TEs (PTEs) and stressful life events (SLEs) were included. For example, studies assessing high levels of stress (viz. perceived stress) in the context of everyday stressors were also included. Measuring trauma exposure without PTSD means that these events are not DSM Criterion A events that cause PTSD and therefore should be considered potentially traumatic events. In our review, studies that assessed for potentially traumatic and stressful life events were included. This should therefore be taken into account when interpreting the findings of these studies. The reliability and validity of the SAQOR tool need to be established. As with other quality assessment tools, the potential impact of differential weighting of the component domains of the SAQOR tool should also be determined. In our use of this instrument, each of the five component domains (sample, control, outcome/exposure, follow‐up, distorting influences, reporting of data) was given equal weighting in the overall quality level assigned. Variations in weighting may be appropriate depending upon the research question.

## 6. Conclusion

The results of the included studies underscore the importance of considering trauma, PTSD, and other common mental health outcomes when conducting research on cognition in PLWHA, as well as when making a diagnosis of HIV associated Neurocognitive Disorders (HAND). In particular, these studies highlight the need for trauma screening over the life course in PLWHA. Stress reduction and post-traumatic stress prevention and treatment programmes could help improve and maintain cognitive function in PLWHA and in turn may contribute to optimal HIV treatment adherence, viral suppression and improved quality of life.
